# Effect of SARS-CoV-2 mRNA vaccination in MS patients treated with disease modifying therapies

**DOI:** 10.1016/j.ebiom.2021.103581

**Published:** 2021-09-22

**Authors:** Maria Pia Sormani, Matilde Inglese, Irene Schiavetti, Luca Carmisciano, Alice Laroni, Caterina Lapucci, Giorgio Da Rin, Carlo Serrati, Ilaria Gandoglia, Tiziana Tassinari, Germana Perego, Giampaolo Brichetto, Paola Gazzola, Antonio Mannironi, Maria Laura Stromillo, Cinzia Cordioli, Doriana Landi, Marinella Clerico, Elisabetta Signoriello, Jessica Frau, Maria Teresa Ferrò, Alessia Di Sapio, Livia Pasquali, Monica Ulivelli, Fabiana Marinelli, Graziella Callari, Rosa Iodice, Giuseppe Liberatore, Francesca Caleri, Anna Maria Repice, Susanna Cordera, Mario Alberto Battaglia, Marco Salvetti, Diego Franciotta, Antonio Uccelli, the CovaXiMS study group on behalf of the Italian Covid-19 Alliance in MS, Alessandro Maglione, Alessia Di Sapio, Alessio Signori, Alice Laroni, Aniello Iovino, Anna Maria Repice, Antonio Mannironi, Antonio Uccelli, Carlo Serrati, Carolina Gabri Nicoletti, Caterina Lapucci, Chiara Rosa Mancinelli, Cinzia Cordioli, Daiana Bezzini, Daniele Carmagnini, Davide Brogi, Diego Franciotta, Doriana Landi, Eduardo Nobile Orazio, Eleonora Cocco, Elisabetta Signoriello, Enri Nako, Ester Assandri, Fabiana Marinelli, Federica Baldi, Filippo Ansaldi, Francesca Bovis, Francesca Caleri, Gabriele Siciliano, Gaia Cola, Germana Perego, Giacomo Lus, Giampaolo Brichetto, Giancarlo Icardi, gianmarco bellucci, Giorgio Da Rin, Girolama Alessandra Marfia, Giulia Vazzoler, Giuseppe Liberatore, Giuseppe Trivelli, Graziella Callari, Ilaria Gandoglia, Ilaria Maietta, Irene Schiavetti, Jessica Frau, Laura Sticchi, Livia Pasquali, Lorena Lorefice, Luca Carmisciano, Lucia Ruggiero, Marcello Manzino, Marco Salvetti, Margherita Monti Bragadin, Maria Chiara Buscarinu, Maria Gagliardi, Maria Laura Stromillo, Maria Pia Sormani, Maria Teresa Ferrò, Maria Teresa Rilla, Marinella Clerico, Mario Alberto Battaglia, Marta Ponzano, Marzia Fronza, Massimo Del Sette, Matilde Inglese, Matteo Scialabba, Michele Bedognetti, Monica Ulivelli, Nicola De Rossi, Nicola De Stefano, Paola Gazzola, Rachele Bigi, Raffaele Dubbioso, Roberta Reniè, Rosa Iodice, Sabrina Fabbri, Sarah Rasia, Simona Rolla, Stefan Platzgummer, Susanna Cordera, Tiziana Tassinari, Valentina Carlini

**Affiliations:** a Department of Health Sciences, Section of Biostatistics, University of Genova, Italy; b IRCCS Ospedale Policlinico San Martino, Genova, Italy; c Department of Neuroscience, Rehabilitation, Ophthalmology, Genetics, Maternal and Child Health (DINOGMI) and Center of Excellence for Biomedical Research (CEBR), University of Genoa, Genoa, Italy; d Laboratory Medicine, IRCCS Ospedale Policlinico San Martino, Genova, Italy; e Department of Neurology, Imperia Hospital, Imperia, Italy; f Neurology Unit, Galliera Hospital; g S.C. Neurologia - Ospedale Santa Corona Pietra Ligure (Sv); h SC Neurologia ASL 4 Chiavarese; i AISM Rehabilitation Center, Genoa, Italy; j Centro Sclerosi Multipla S.C. Neurologia Asl 3 Genovese; k Department of Neurology, Sant'Andrea Hospital, La Spezia, Italy; l Clinica Neurologica e Malattie Neurometaboliche, Università degli Studi di Siena; m Centro Sclerosi Multipla ASST Spedali Civili di Brescia; n Multiple Sclerosis Clinical and Research Unit, Department of Systems Medicine, Tor Vergata University and Hospital, Rome, Italy; o Dipartimento di Scienze Cliniche e Biologiche, Università di Torino Università di Torino; p Centro Sclerosi Multipla, II Clinica Neurologica, Università della Campania Luigi Vanvitelli; q Centro Sclerosi Multipla Ospedale Binaghi Cagliari - ATS Sardegna, Università di Cagliari; r Neuroimmunology, Center for Multiple Sclerosis, Cerobrovascular Department, Neurological Unit, ASST Crema; s Department of Neurology, Regina Montis Regalis Hospital, Mondovì, Italy; t Department of Clinical and Experimental Medicine, Neurology Unit, University of Pisa, Italy; u Department of Medicine, Surgery and Neuroscience, University of Siena; v Multiple Sclerosis Center, Fabrizio Spaziani Hospital, via Armando Fabi, Frosinone, Italy; w UOC Neurologia e Centro SM Fondazione Istituto G. Giglio, Cefalù; x Clinica Neurologica, DSNRO Università Federico II di Napoli; y Neuromuscular and Neuroimmunology Service, IRCCS Humanitas Research Hospital, Rozzano, Italy; z MS Center, Department of Neurology, F. Tappeiner Hospital Meran (BZ), Italy; aa Department of Neurology 2, Careggi University Hospital, Florence, Italy; ab Department of Neurology, Ospedale Regionale, Aosta, Italy; ac Research Department, Italian Multiple Sclerosis Foundation, Genoa, Italy; ad Department of Life Sciences, University of Siena, Italy; ae Centre for Experimental Neurological Therapies (CENTERS), Department of Neurosciences, Mental Health and Sensory Organs, Sapienza University of Rome, Italy; af IRCCS Istituto Neurologico Mediterraneo Neuromed, Pozzilli, Italy; ag Autoimmunology Laboratory, IRCCS Ospedale Policlinico San Martino, Genoa, Italy

**Keywords:** Multiple sclerosis, Coronavirus, Immunomodulatory therapies

## Abstract

**Background:**

In patients with Multiple Sclerosis (pwMS) disease-modifying therapies (DMTs) affects immune response to antigens. Therefore, post-vaccination serological assessments are needed to evaluate the effect of the vaccine on SARS-CoV-2 antibody response.

**Methods:**

We designed a prospective multicenter cohort study enrolling pwMS who were scheduled for SARS-Cov-2 vaccination with mRNA vaccines (BNT162b2, Pfizer/BioNTech,Inc or mRNA-1273, Moderna Tx,Inc). A blood collection before the first vaccine dose and 4 weeks after the second dose was planned, with a centralized serological assessment (electrochemiluminescence immunoassay, ECLIA, Roche-Diagnostics). The log-transform of the antibody levels was analyzed by multivariable linear regression.

**Findings:**

780 pwMS (76% BNT162b2 and 24% mRNA-1273) had pre- and 4-week post-vaccination blood assessments. 87 (11·2%) were untreated, 154 (19·7%) on ocrelizumab, 25 (3·2%) on rituximab, 85 (10·9%) on fingolimod, 25 (3·2%) on cladribine and 404 (51·7%) on other DMTs. 677 patients (86·8%) had detectable post-vaccination SARS-CoV-2 antibodies. At multivariable analysis, the antibody levels of patients on ocrelizumab (201-fold decrease (95%CI=128–317), *p* < 0·001), fingolimod (26-fold decrease (95%CI=16–42), *p <* 0·001) and rituximab (20-fold decrease (95%CI=10–43), *p <* 0·001) were significantly reduced as compared to untreated patients. Vaccination with mRNA-1273 resulted in a systematically 3·25-fold higher antibody level (95%CI=2·46–4·27) than with the BNT162b2 vaccine (*p <* 0·001). The antibody levels on anti-CD20 therapies correlated to the time since last infusion, and rituximab had longer intervals (mean=386 days) than ocrelizumab patients (mean=129 days).

**Interpretation:**

In pwMS, anti-CD20 treatment and fingolimod led to a reduced humoral response to mRNA-based SARS-CoV-2 vaccines. As mRNA-1273 elicits 3·25-higher antibody levels than BNT162b2, this vaccine may be preferentially considered for patients under anti-CD20 treatment or fingolimod. Combining our data with those on the cellular immune response to vaccines, and including clinical follow-up, will contribute to better define the most appropriate SARS-CoV-2 vaccine strategies in the context of DMTs and MS.

**Funding:**

FISM[2021/Special-Multi/001]; Italian Ministry of Health‘Progetto Z844A 5 × 1000′.

**Research in context**

**Evidence before this study**

We searched PubMed (from year 2000) for cohort observational studies and randomized trials assessing the effect of vaccine in patients with MS under DMTs. (Search terms: “Multiple Sclerosis and Disease Modifying Therapies and Vaccine” in the Title or Abstract). We found a recently published meta-analysis (Ciotti et al., MSRAD, October 2020) summarizing the evidence about the effects of MS disease-modifying therapies on responses to vaccinations. This meta-analysis included 16 independent studies, mostly focused on humoral responses, with few examining cellular immune responses to vaccination. Several studies demonstrated preserved immune responses in people treated with interferon. A few studies also suggested a preserved vaccine response in patients on dimethyl fumarate. Vaccine responses were reduced to varying degrees in those treated with glatiramer acetate, teriflunomide, sphingosine-1-phosphate receptor modulators, and natalizumab. The timing of vaccination played an important role in those treated with alemtuzumab. Humoral vaccine responses were significantly impaired by anti-CD20 monoclonal antibody therapies. Data were lacking on vaccine responses in patients on cladribine. A study on response to vaccination in patients on ocrelizumab was subsequently published, confirming an impaired response in these patients (VELOCE study). Recently, 2 small studies (*n* = 125 and *n* = 32 respectively) reported on humoral response to Sars-Cov-2 vaccines in people treated with fingolimod (positivity in 1 over 26 and in 10 over 16 respectively) cladribine (23 over 23), ocrelizumab (10 over 37 and 6 over 16 respectively).

**Added value of this study**

This study reports on the humoral response to Sars-Cov-2 mRNA vaccines of a large sample of patients with MS treated with all the DMTs. The impaired response to Covid-19 mRNA vaccines is firmly confirmed in patients under anti-CD20 therapies and on fingolimod. The antibody levels 4 weeks after vaccination increase in people treated with anti-CD20 therapies with longer interval between the last infusion of the drug and the first dose of vaccine. Interestingly, the antibody titers after vaccination with mRNA-1273 (Moderna) are significantly higher than the antibody titers after vaccination with BNT162b2 (Pfizer/BioNTech).

**Implications of all the available evidence**

This finding can have an impact on the choice of the vaccine for patients treated with anti-CD20 and fingolimod, even if additional information on cellular response is needed to refine the vaccination strategy in these patients.

Alt-text: Unlabelled box

## 1 Introduction

The National Multiple Sclerosis Society and other expert organizations recommended that all patients with multiple sclerosis (pwMS) should be vaccinated against SARS-CoV-2. The impact of such vaccination, mainly in terms of serological responses, adverse effects, and clinical effectiveness, on pwMS treated with disease-modifying therapies (DMTs) is largely unknown. Preliminary results on safety and immunogenicity of vaccination by the lipid nanoparticle-formulated BNT162b2 Covid-19 vaccine (Pfizer-BioNTech) came from Israel. A first paper, reporting about 555 patients who received their first dose and 453 who received their second dose, showed that the rate of acute relapses following the first and second doses was similar to the rate in non-vaccinated patients during the corresponding period, reassuring about the possibility that vaccination may drive pathogenic immune responses that can trigger disease reactivation [1]. In a second paper [2], the authors reported about SARS-CoV-2 IgG response one month after the second dose using anti-spike protein-based serology in 125 MS patients under different DMTs vaccinated with BNT162b2 Covid-19 vaccine. They observed that only 22·7% of patients treated with ocrelizumab developed IgG response irrespective of normal absolute lymphocyte count, that most fingolimod-treated MS patients failed to develop SARS-COV-2 antibodies and that cladribine treatment had little impact on humoral response to Covid-19 vaccine. A small study on 32 pwMS was recently published, indicating impaired antibody response to BNT162b2 Covid-19 vaccine during fingolimod and ocrelizumab treatment [3].

This pilot study was designed at the start of the vaccination campaign in Italy, to monitor side effects and immunogenicity of vaccine against SARS-Cov-2 in pwMS vaccinated with mRNA vaccines, that were indicated for frail patients by the regulatory agency, namely Agenzia Italiana del Farmaco (AIFA).

## 2 Methods

### 2.1 Study design and participants

This observational multi-center prospective study was conducted in 35 Italian MS centers on pwMS undergoing the SARS-CoV-2 vaccination. Adult pwMS, with or without a previous SARS-CoV-2 infection who were scheduled for SARS-CoV-2 vaccination, were included in the study. mRNA vaccines (BNT162b2 (Pfizer Inc, and BioNTech) or mRNA-1273 (Moderna Tx, Inc)) as per clinical practice and regional indications were allowed. Main reason for study exclusion were the presence of known allergic reactions to components of the vaccine and/or any relevant comorbidities requiring additional treatments with B-cell–targeted therapies, lymphocyte-trafficking blockers, alemtuzumab, anti-CD4 antibody, cladribine, cyclophosphamide, mitoxantrone, azathioprine, mycophenolate mofetil, cyclosporine, methotrexate, total body irradiation, or bone marrow transplantation. Patients who agreed to provide a first blood test sample just before the vaccination and a second drawing one month after the last dose were enrolled in the study. A follow up study with an additional blood test after 6 months and a final safety assessment after approximately 18 months were scheduled.

Here, we report of an interim data analysis on immunogenicity of the vaccination, related to a first subgroup of patients who have already received two vaccine doses over the 2000 patients planned for enrollment in the study.

### 2.2 Ethics

The study is done in compliance with the principles of the Declaration of Helsinki. The protocol is approved by the regional (CER Liguria: 5/2021 - DB id 11169- 21/01/2021) and the centralized national ethical committee AIFA/Spallanzani (Parere n 351, 2020/21). Written informed consent was obtained from all participants before starting any study procedures.

#### 2.2.1 Study procedures

Eligible subjects were contacted by their neurologist before receiving the first vaccine dose against Covid-19 and were informed about the study design and aims. Patients who agreed to participate, after signing a written informed consent, underwent a routine neurological visit where all demographical and medical history data were recorded. Subsequently, a first blood sample was collected to assess antibody levels before vaccination (within one month). A telephone call between the first and the second vaccination dose was made for monitoring the occurrence of any relevant safety issues.

Finally, after four weeks from the second dose, MS patients underwent another neurological visit, and a second blood sample was collected to test post-vaccine antibody levels.

#### 2.2.2 Assessment of antibody responses

High-affinity pan-Ig antibodies to SARS-CoV-2 were measured by a centralized laboratory with a double-antigen sandwich-based electrochemiluminescence immunoassay (ECLIA), using commercial kits (Elecsys®, Roche Diagnostics Ltd, Switzerland). We quantitatively measured receptor-binding domain (RBD) antibodies (Anti-SARS-CoV-2 S), to evaluate the humoral immune response to the two RBD-coding mRNA vaccines, and Nucleocapsid (N) antibodies (Anti-SARS-CoV-2 N), to evaluate previous/coincident responses to the natural infection. RBD antibodies (Anti-SARS-CoV-2 S) have been shown to positively correlate with SARS-CoV-2 neutralizing antibodies on neutralization assays [4,5]. Serum samples were shipped in dried ice by the centers and stored at −20 °C until analysis.

#### 2.2.3 Primary outcome: humoral immunogenicity

The primary objective of this interim analysis was to quantify the levels of SARS-CoV-2 RBD antibodies elicited by vaccination, according to DMT exposure. The cut-off of positivity was 0·80 U/mL for RBD antibodies (response to the vaccine), and 1·0 COI (cut-off index) for N antibodies (seropositivity due to natural infection), in accordance with the manufacturer's instructions. In pwMS with antibody positivity for RBD at pre-vaccination samples, positive response to the vaccine was set at equal or more than 4-fold increase in RBD antibody levels.

#### 2.2.4 Statistical analysis

We planned to evaluate 2000 MS patients, with an interim analysis to assess immunogenicity after about 750 samples. To have a power of 90% to detect a difference in the antibody titer between at least two groups treated with different DMTs of 1 log_10_ unit [6], with 12 DMT groups (alpha level=0·0041 with Bonferroni correction) and a standard deviation (SD) of 1·2 log_10_ unit [6], an average number of 52 MS patients per DMT group was needed, for a total of 624 patients. To allow for a 20% of missing values we planned to run the interim immunogenicity analysis after the first 748 patients enrolled.

The percentage of patients who had a positive serological test before vaccination is reported for all the patients who had the first blood assessment. All the analyses to assess antibody levels against vaccine were run on patients who received two vaccination doses.

The antibody levels were transformed on a Log10 scale, to normalize their distribution and according to previous literature [6]. A linear regression model was used to compare the antibody titers across patients treated with different DMTs, after adjusting for age, sex, BMI, EDSS level, disease duration, presence of comorbidities, antibody levels in the pre-vaccination samples and vaccine type. The effect on the post-vaccination antibody levels of all the relevant covariates was expressed as a geometric mean, that represents the multiplicative factor for the reference level of the considered covariate. We also checked for a different treatment effect according to vaccine type by inserting in the model a treatment by vaccine type interaction. To maximize the efficient use of the available data we used an advanced multiple imputation of missing values strategy (10 imputations) for missing baseline data [7]. We run a sensitivity analysis including patients with complete information. The same multivariable model, adjusting for the days since last infusion was run on patients on anti-CD20 therapies, to check for a difference of antibody levels between rituximab and ocrelizumab.

The relationship between antibody levels after vaccination and the time since last infusion of an anti-CD20 agent (rituximab or ocrelizumab) was assessed by a non-linear fit with a Gompertz growth curve. The correlation between antibody levels after vaccination and lymphocyte counts for patients in therapy with fingolimod was assessed by a linear model after adjusting for pre-vaccination positivity and vaccine type.

### 2.3 Role of the funding sources

The study was funded by FISM [2021/Special-Multi/001] and by the Italian Ministry of Health ‘Progetto Z844A 5 × 1000′. The funding sources did not have any role in data analysis and interpretation.

## 3 Results

### 3.1 Pre-vaccination serological positivity

Data were collected between March 4, 2021 and July 9, 2021. At the time of the interim analysis 1202 pwMS have been invited to participate in the study. Of them 1022 (85%) accepted to participate. Among the 180 who refused, 29 (16%) declined the vaccination and 151 (84%) did not want to come for the blood sampling. At the time of interim analysis, we assessed antibody levels of 1022 patients before vaccination. Of them 114 (11·2%) were positive for RBD, N, or both antibodies. Among them, just 38 (33·3%) reported a previous SARS-CoV-2 infection (they responded “yes” to the question: “Did you have a prior confirmed SARS-CoV-2 infection?”), indicating that 66·7% of seropositive patients were unaware of their past SARS-CoV-2 infection.

### 3.2 Post-vaccination results

780/1022 (76%) patients had the blood sample assessed for RBD and for N antibodies 4 weeks after vaccination (mean time after vaccination 33 days (SD=8 days)) and their characteristics are reported in Table 1. All the patients received two vaccine doses. 594 patients (76·2%) were vaccinated with BNT162b2 and 186 patients (23·8%) with mRNA-1273.

**Table 1:** Baseline demographic and clinical characteristics of the included patients.

Characteristic	Patients who received two vaccine doses (*n* = 780)
Age − Mean (SD)	45·8 (12)
Female sex − no. (%)	517 (66·3)
BMI − Mean (SD)	24·3 (4·9)
Missing (%)	166 (21·3)
MS phenotype − no. (%)	
Primary progressive	73 (9·4)
Relapsing remitting	637 (81·7)
Secondary progressive	70 (9·0)
MS disease duration (yr) − Median (IQR)	9·4 [0·2, 54·9]
Missing (%)	8 (1·0)
EDSS − Median (IQR)	2·0 [1·0, 3·6]
Missing (%)	3 (0·4)
MS Treatment − no. (%)	
Dimethyl-fumarate	114 (14·6)
Fingolimod	85 (10·9)
Ocrelizumab	154 (19·7)
Natalizumab	100 (12·8)
Interferon	79 (10·1)
Glatiramer-Acetate	38 (4·9)
Teriflunomide	48 (6·2)
Alemtuzumab	15 (1·9)
Cladribine	25 (3·2)
Rituximab	25 (3·2)
Other	10 (1·3)
None	87 (11·2)
Positivity for RBD, N, or both antibodies before vaccination	73 (9·4)
Prior Covid − no. (%)	35 (4·5)
Missing (%)	27 (3·5)
Vaccine type − no. (%)	
BNT162b2	594 (76·2)
mRNA-1273	186 (23·8)

SD= Standard deviation, IQR=Inter-quartile range. Where the number of missing values is not reported there are no missing values.

Among the 780 vaccinated patients, 73 (9·4%) were positive for RBD, N, or both antibodies before vaccination and 677 (86·8%) were positive for RBD post-vaccination. 68 out of 73 patients (93·1%) who were positive at the pre-vaccination test responded to vaccine with a post-vaccination >4-fold increase in RBD antibody levels.

**Fig. 1:**
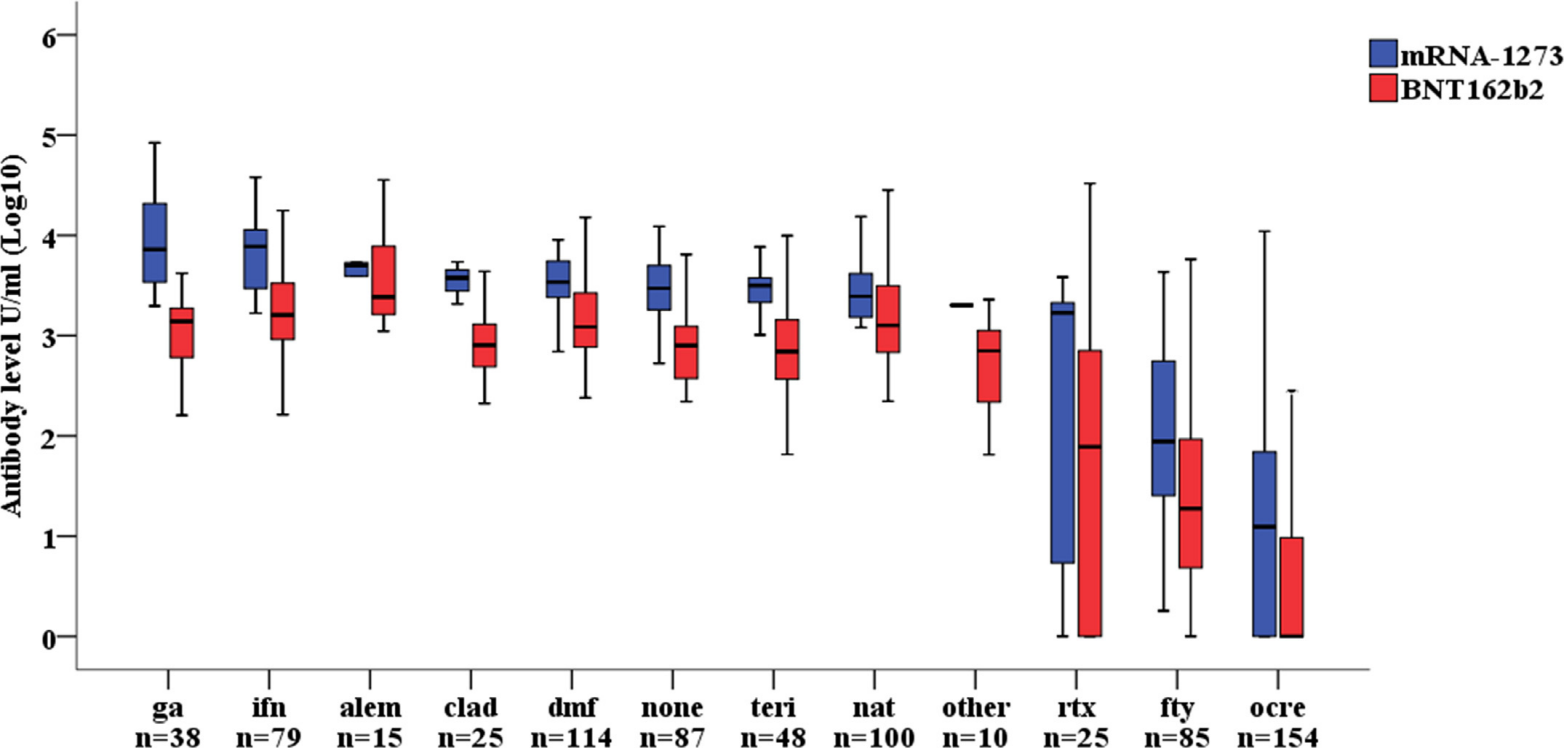
Post-vaccination RBD antibody levels by disease modifying treatment in relation to vaccine type. Footnote: ga=glatiramer-acetate, ifn=interferon, alem=alemtuzumab, clad=cladribine, dmf=dimethyl-fumarate, teri=teriflunomide, rtx=rituximab, fty=fingolimod, ocre=ocrelizumab.

The post-vaccination RBD antibody levels in each DMT group and according to vaccine type (BNT162b2 or mRNA-1273) is reported in Fig. 1. All patients mounted a full response to the vaccine as depicted by positive antibody levels against RBD, excluding one patient treated with interferon (1·3%), 6 patients treated with fingolimod (7·1%), 87 patients treated with ocrelizumab (56·5%) and 9 patients treated with rituximab (36·0%).

The results of the multivariable regression are reported in Table 2.

**Table 2:** Multivariable analysis assessing factors associated to antibody levels, 4 weeks after the full vaccination course.

	Multivariable Analysis		
Variable	Beta coefficients (SE)	Geometric mean* (95% CI)	p
Age (10 years)	−0·04 (0·03)	0·91 (0·80–1·04)	0·16
Sex (Female vs Male)	0·03 (0·06)	1·06 (0·83–1·37)	0·63
MS type (Progressive vs RR)	0·08 (0·09)	1·21 (0·81–1·79)	0·35
EDSS	−0·02 (0·02)	0·96 (0·88–1·05)	0·36
Disease duration>10 years	−0·08 (0·06)	0·83 (0·64–1·06)	0·14
Comorbidities (yes/no)	0·05 (0·06)	1·12 (0·86–1·47)	0·42
BMI	0·01 (0·01)	1·03 (0·99–1·06)	0·06
Pre-vaccination antibody level (log10)	0·90 (0·09)	4·11 (3·23–5·28)	<0·001
Vaccine type			
BNT162b2	Ref		
mRNA-1273	0·51 (0·06)	3·25 (2·46–4·27)	<0·001
Disease modifying therapy			<0.001
No therapy**	Ref		
Interferon	0·25 (0·11)	1·77 (1·06–2·96)	
Glatiramer-Acetate	0·27 (0·14)	1·85 (0·99–3·47)	
Teriflunomide	0·02 (0·13)	1·04 (0·58–1·87)	
Dimethyl-fumarate	0·16 (0·11)	1·43 (0·88–2·31)	
Natalizumab	0·06 (0·11)	1·16 (0·70–1·93)	
Fingolimod	−1·41 (0·11)	0·039 (0·023–0·064)#	<0·001**
Ocrelizumab	−2·30 (0·10)	0·005 (0·003–0·008)#	<0·001**
Rituximab	−1·31 (0·16)	0·049 (0·024–0·102)#	<0·001**
Cladribine	−0·04 (0·17)	0·92 (0·44–1·94)	
Alemtuzumab	0·45 (0·20)	2·84 (1·15–7·03)	
Other	−0·14 (0·24)	0·73 (0·25–2·12)	

*The geometric mean represents the multiplicative factor of each level of the variable as compared to the reference level.

**Disease modifying therapy had a significant p value, indicating significant heterogeneity of antibody levels among the different therapies. No therapy was chosen as the reference class to express the beta coefficients and the geometric means. In the multivariable model only the p-values of 3 disease modifying therapies that are significantly different from all the others after a post-hoc analysis with Bonferroni correction are reported.

# The effect of fingolimod is a 26-fold decrease (95%CI=16–43), the effect of ocrelizumab was a 201-fold decrease (128–317) and the effect of rituximab was a 20-fold decrease (10–43).

The factors significantly associated to post-vaccination antibody titers were the pre-vaccination antibody level (with a 4·11-fold increase (95%CI=3·23–5·28) every log_10_ unit baseline increase, *p <* 0·001), type of vaccine (with mRNA-1273 giving a 3·25-fold higher (95%CI=2·46–4·27) RBD antibody levels than BNT162b2 (*p <* 0·001)) and the DMT used by the patient: among the DMT groups, patients treated with ocrelizumab (with a 201-fold decrease (95%CI=128–317) (*p <* 0·001), fingolimod (with a 26-fold decrease (95%CI=16–42), *p <* 0·001), and rituximab (with a 20-fold decrease (95%CI=10–43), *p <* 0·001) showed significantly reduced RBD antibody levels as compared to untreated patients. No other differences were detected among all the other DMTs. Missing data were mostly on BMI (*n* = 166, 21·3%) and the sensitivity analysis run on patients with complete information (*n* = 614, 78·7%) gave the same results.

The percentage of patients on fingolimod, ocrelizumab and rituximab with antibody levels above the cut-off of positivity was 100% (21/21), 61% (14/23) and 71% (5/7) respectively among those vaccinated with mRNA-1273 vs 90·6% (58/64), 40·5% (53/131) and 61% (11/18) among those vaccinated with BNT162b2.

**Fig. 2:**
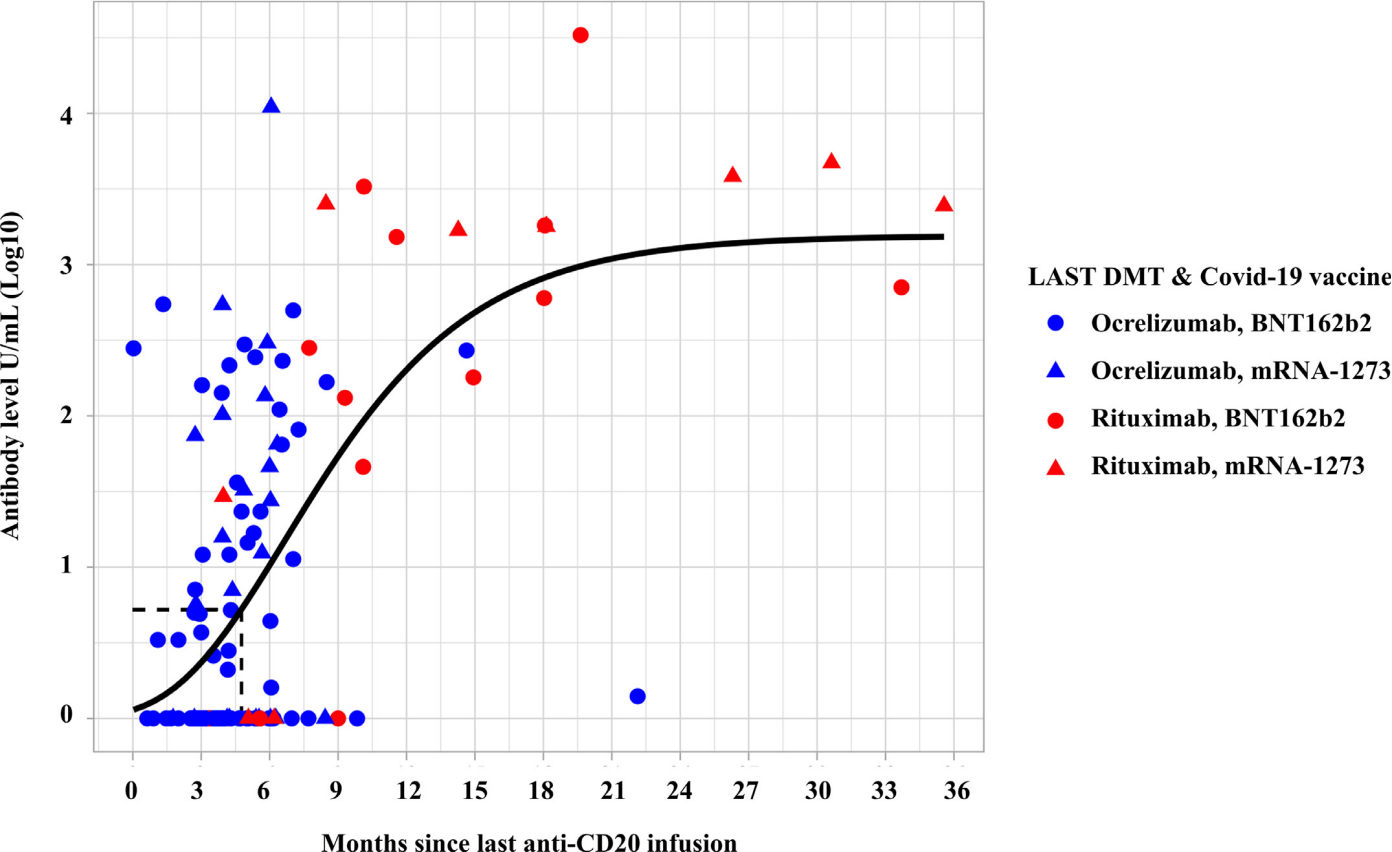
Post-vaccination RBD antibody levels in patients treated with anti-CD20 therapies according to the time passed since the last infusion.

For patients who were on therapy with an anti-CD20 agent (ocrelizumab or rituximab) we evaluated RBD antibody levels 4 weeks after the last vaccine dose as a function of the time passed between the last infusion of the drug and the first dose of vaccine. As shown in Fig. 2, there was a progressive increase of the RBD antibody levels in response to vaccine with an increasing interval from the last anti-CD20 infusion. The median time since last infusion was significantly higher for patients on rituximab (386 days, range=100–1011 days) than for patients on ocrelizumab (129 days, range=19–439 days) (*p <* 0·001, Fig. 2). The inflection point of the curve is at day 143 (95%CI=84–258), indicating that in these patients at least this time spam between the last infusion and the vaccination is necessary to have an antibody response to vaccine. The antibody levels in patients receiving ocrelizumab was non-significantly lower (1·6-fold reduction) than the antibody levels of patients receiving rituximab, after adjusting for the time passed between the last infusion of the drug and the first dose of vaccine (geometric mean=0·63, 95%CI=0·35–1·13, *p* = 0·12). On the other hand, the antibody levels were significantly associated with the time since last dose (geometric mean per month: 1·12 (95%CI=1·07–1·17, *p <* 0·001).

**Fig. 3:**
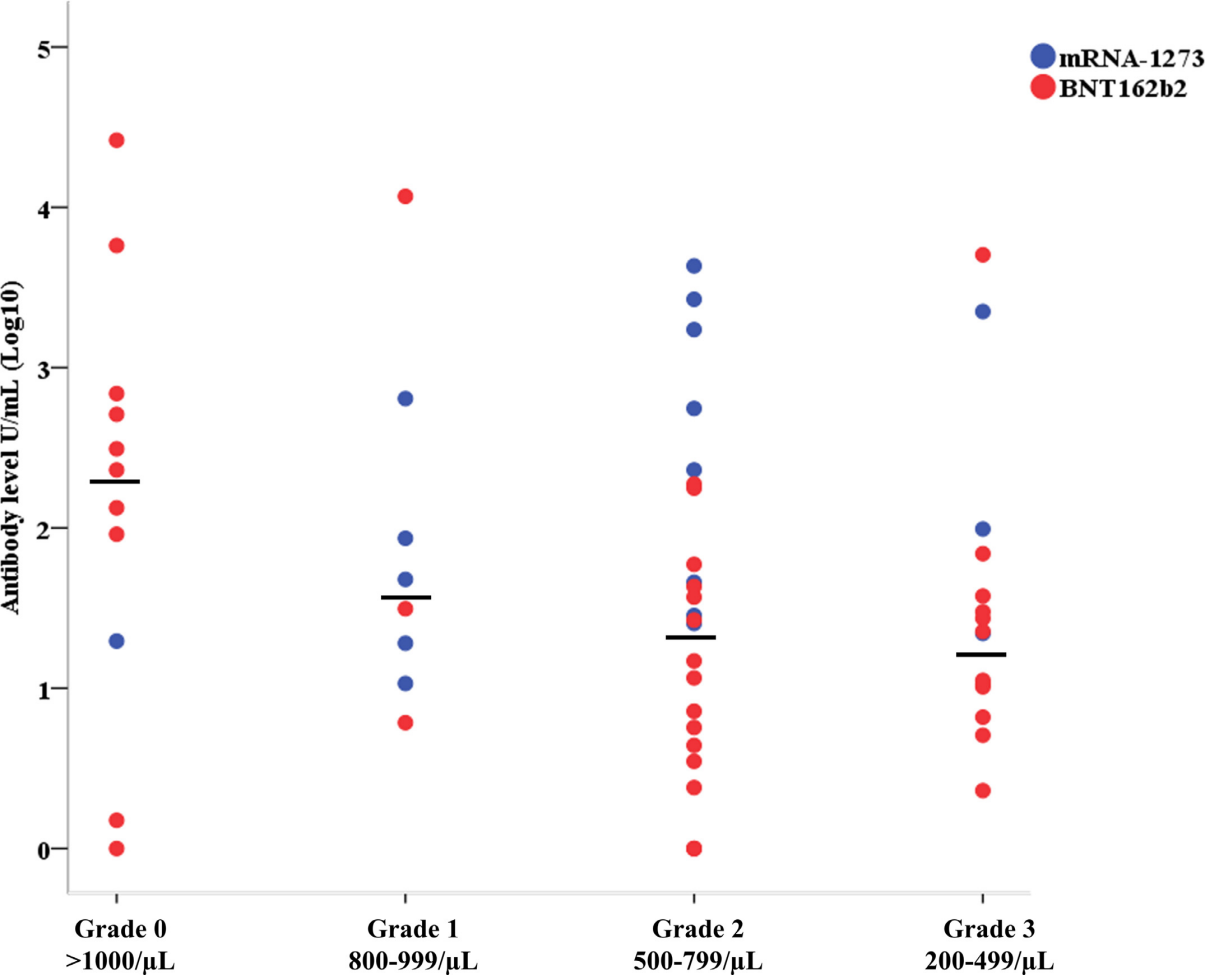
Anti-SARS-CoV-2 RBD antibody titers in patients treated with fingolimod according to their lymphocyte counts (measured within one month since the first dose of vaccine).

Among the 85 patients on therapy with fingolimod 57 had complete information on lymphocyte counts: we examined the relationship of RBD antibody levels with the degree of lymphopenia assessed within one month from the first dose of vaccine. We observed a significant decrease of RBD antibody levels associated with presence of lymphopenia (lymphocyte counts <1000/µL) (*p* = 0·034, Fig. 3), after adjusting for pre-vaccination positivity and vaccine type. Despite the small sample size of this group, patients vaccinated with mRNA-1273 showed significantly higher RBD antibody levels (geometric mean 10·1 (95%CI=3·7–32·3), *p <* 0·001).

## 4 Discussion

In this study we measured the vaccine-specific total immunoglobulin response to the RBD of SARS-CoV-2 Spike 1 protein, the main target of serum neutralizing activity [8], in a group of pwMS on different types of DMTs. All the patients in this cohort were vaccinated with mRNA vaccines (either mRNA-1273 or BNT162b2). Post-vaccination humoral responses resulted impaired in MS patients treated with ocrelizumab and rituximab and, to a lesser extent, with fingolimod. 56·5% of vaccinated patients on ocrelizumab, 36·0% of vaccinated patients on rituximab and 7·1% of vaccinated patients in fingolimod failed to produce detectable RBD antibody levels 4 weeks after the second vaccination dose. This time-point, which should coincide with the highest levels of antibody production, has been chosen considering previous data on vaccine humoral responses in pwMS on B cell-depleting drugs [9].

As compared to untreated patients, the median RBD antibody levels of patients on ocrelizumab were reduced by a factor of 201, of patients on rituximab by a factor of 20 (though the difference between the two antiCD20 are related to time from infusion), and of patients under fingolimod by a factor of 26.

These findings substantially confirm and expand what previously reported on the partial lack of antibody response to the BNT162b2 vaccine in those on ocrelizumab and on fingolimod [1,3] and on the full response in those on cladribine [1]. From a mechanistic point of view, if the negative influence on antibody responses to vaccines by B cell-depleting therapies was expected [9], the effects of fingolimod on such responses was less expected and might be associated with its inhibition of germinal center reaction [10]. The subsequent impairment of plasma antibody production preferentially affects T-dependent antigens [11]. Fingolimod-treated patients can mount immune responses to novel and recall antigens, but with reduced response and seroconversion rates [12]. In our series, the rate of seroconversion was very high, namely, 100% in pwMS vaccinated with mRNA-1273, and 90·6% in those vaccinated with BNT162b2, but the antibody levels that guarantee vaccine clinical protection are unknown at present.

As a surprising finding of our study, RBD antibody levels, were 3·25 times higher in patients who received the mRNA-1273 vaccine versus those who were vaccinated with BNT162b2 vaccine. Both vaccines use pseudo-uridine in place of uridine to try to circumvent the well-known inflammatory reactions to foreign mRNA and, on the other hand, they contain mRNA wrapped in lipid nanoparticles that can act as adjuvants, thus bolstering the immune response. However, while each dose of BNT162b2 contains 30 micrograms of vaccine, mRNA-1273 contains 100 micrograms: clinical effectiveness was similar, whereas MRNA-1273 vaccine associated with a greater incidence of adverse events [13]. Analogously, it is likely that the higher mRNA content could also account for the higher RBD antibody levels versus BNT162b2 that we observed in our case series. A higher production of RBD protein could additionally contribute to the overall immunogenic effect. As for other settings, mRNA-1273 and BNT162b2 vaccines yielded similar percentages of positive RBD antibody response in dialysis patients, but only 18/168 of them were administered with mRNA-1273 vaccine [14]. Analogous findings were reported in mRNA-1273/BNT162b2 vaccinated rhesus macaques [15], and in 20 volunteers tested for antibody neutralizing activity [16]. Conversely, among pregnant women, higher antibody titers were found in those vaccinated with mRNA-1273 vaccine [17]^(pre-print)^. Our data, obtained on a very large sample size, warrant confirmation on healthy populations.

Finally, RBD antibody levels progressively increased in patients on anti-CD20 therapies in correlation with the time passed since last infusion to the first vaccination dose, with 143 days as the time-point when the antibody levels start to increase. Analogously, RBD antibody levels in patients on fingolimod seems to depend on lymphocyte counts and are higher after vaccination with mRNA-1273 vaccine, in line with what above-mentioned on the drug's mechanistic effects on the immune system.

### 4.1 Caveats and limitations

This study has limitations related to observational study design and its selection bias and possible unmeasured confounding. Also, there are limitations in a-priori decisions on the study design: for example, it is not known whether 4-week time point is optimal for assessing response. In the absence of consistent data when we planned the project, we referred to a study on pharmacologically B-cell depleted MS patients who received influenza vaccine, and whose virus-specific antibody titer peaked 4 weeks after single dose vaccinations [9]. Recent findings, however, suggest that SARS-CoV-2 antibody responses tend to be the highest 8 weeks after the second dose [16]. Finally, since frail patients in Italy were vaccinated with mRNA vaccines only, this study has no data on adenovirus-based vaccines.

Clinical effectiveness of a vaccination does not exclusively depend on the humoral immune response, especially in the case of coronaviruses, against which cellular immune responses play an important role [13]. The reported mRNA SARS-CoV-2 clinical trials have noted increases in total antibodies, neutralizing antibodies (NAb), and T-cell responses in healthy controls following vaccination [10] and immunological studies in MS patients on ocrelizumab after Covid-19 infection reported the absence of virus-specific antibodies, but valid T-cell responses [18]. Yet to be peer-reviewed, one study showed that all the MS patients on anti-CD20 therapies, and vaccinated with mRNA vaccines, generated antigen-specific CD4+ and CD8+ T-cell responses [19]^(pre-print)^, and another one, on the contrary, that such responses were detectable in 17% of patients and in 86% of healthy controls only [20]^(pre-print)^. It is likely that the discrepancies are due to sensitivity in the T-cell assays. The levels of T-cell-mediated protection from Covid-19 in patients on B-cell depleting therapies will be evaluable in the future, knowing that, in contrast to neutralizing antibodies, SARS-CoV-2-specific CD4 and CD8 T lymphocytes associate with milder disease course, suggestive of protective immunity [21], [22]. However, it is still unclear which combination of the immune responses is responsible for the best immunity to the virus, both in healthy subjects and in patients on B and T cell-depleting therapies.

Moreover, we lack the information on the quantitative levels of virus-specific antibodies following vaccination that can guarantee a clinically effective protection against the infection. As a first step towards standardization of the antibody reports, World Health Organization established an International Standard for SARS-CoV-2 antibodies, assessing the neat sample to contain 1000 binding antibody units (BAU)/mL [23]. A mathematical transposition of Elecsys-S Units to BAU (Elecsys-S Units = 0·972 x BAU) [6] indicates that Elecsys-S U/mL can be considered substantially equivalent to BAU/mL. Even in the absence of information on the protective levels of SARS-CoV-2 antibodies, the better ability of mRNA-1273 vaccination to boost the antibody levels versus the BNT162b2 vaccine, circumstantially suggests that the former might be preferred in frail people. This category includes subjects that diseases, therapies, age, or a combination of these factors, can affect their responses to SARS-CoV-2. Our data on post-vaccination decreasing RBD antibody levels, without correlation for gender and body-mass index, is also in line with what reported on SARS-CoV-2 neutralizing antibodies, and on the demographic-constitutional factors [24].

Overall, the results reported in this study must be interpreted with caution when trying to derive their clinical implications. Profiling T cell-mediated responses to SARS-CoV-2 vaccination, together with clinical follow-up, will add important information aimed at defining the most appropriate SARS-CoV-2 vaccine strategies in the setting of DMTs and MS.

## CovaXiMS study group

**Table:** 

Authors	Affiliation

Alessandro Maglione	Dipartimento di Scienze Cliniche e Biologiche, Università di Torino Università di Torino
Alessia Di Sapio	Department of Neurology, Regina Montis Regalis Hospital, Mondovì, Italy
Alessio Signori	Department of Health Sciences, Section of Biostatistics, University of Genova, Italy
Alice Laroni	Department of Neuroscience, Rehabilitation, Ophthalmology, Genetics, Maternal and Child Health (DINOGMI) and Center of Excellence for Biomedical Research (CEBR), University of Genoa, Genoa, Italy IRCCS Ospedale Policlinico San Martino, Genova, Italy
Aniello Iovino	Clinica Neurologica, DSNRO Università Federico II di Napoli
Anna Maria Repice	Department of Neurology 2, Careggi University Hospital, Florence, Italy
Antonio Mannironi	Department of Neurology, Sant'Andrea Hospital, La Spezia, Italy
Antonio Uccelli	Department of Neuroscience, Rehabilitation, Ophthalmology, Genetics, Maternal and Child Health (DINOGMI) and Center of Excellence for Biomedical Research (CEBR), University of Genoa, Genoa, Italy IRCCS Ospedale Policlinico San Martino, Genova, Italy
Carlo Serrati	Department of Neurology, Imperia Hospital, Imperia, Italy
Carolina Gabri Nicoletti	Multiple Sclerosis Clinical and Research Unit, Department of Systems Medicine, Tor Vergata University and Hospital, Rome, Italy
Caterina Lapucci	Department of Neuroscience, Rehabilitation, Ophthalmology, Genetics, Maternal and Child Health (DINOGMI) and Center of Excellence for Biomedical Research (CEBR), University of Genoa, Genoa, Italy IRCCS Ospedale Policlinico San Martino, Genova, Italy
Chiara Rosa Mancinelli	Centro Sclerosi Multipla ASST Spedali Civili di Brescia
Cinzia Cordioli	Centro Sclerosi Multipla ASST Spedali Civili di Brescia
Daiana Bezzini	Department of Life Sciences, University of Siena
Daniele Carmagnini	Centro Sclerosi Multipla Ospedale Binaghi Cagliari - ATS Sardegna, Università di Cagliari
Davide Brogi	S.C. Neurologia - Ospedale Santa Corona
Diego Franciotta	Autoimmunology Laboratory, IRCCS Ospedale Policlinico San Martino, Genoa, Italy
Doriana Landi	Multiple Sclerosis Clinical and Research Unit, Department of Systems Medicine, Tor Vergata University and Hospital, Rome, Italy
Eduardo Nobile Orazio	Neuromuscular and Neuroimmunology Service, IRCCS Humanitas Research Hospital, Rozzano, Italy Department of Medical Biotechnology and Translational Medicine, Milan University, Milan, Italy
Eleonora Cocco	Centro Sclerosi Multipla Ospedale Binaghi Cagliari - ATS Sardegna, Università di Cagliari
Elisabetta Signoriello	Centro Sclerosi Multipla, II Clinica Neurologica, Università della Campania Luigi Vanvitelli
Enri Nako	Department of Neurology, Regina Montis Regalis Hospital, MondovÃ¬, Italy
Ester Assandri	Neuroimmunology, Center for Multiple Sclerosis, Cerobrovascular Department, Neurological Unit, ASST Crema
Fabiana Marinelli	Multiple Sclerosis Center, Fabrizio Spaziani Hospital, via Armando Fabi, Frosinone – Italy
Federica Baldi	Department of Neurosciences, Rehabilitation, Ophthalmology, Genetics, Maternal and Child health, University of Genova, Genova, Italy
Filippo Ansaldi	Planning, Epidemiology and Prevention Unit, A.Li.Sa. Liguria Health Authority, Genoa, Italy. IRCCS San Martino Hospital, Genoa, Italy. Department of Health Sciences, University of Genoa, Genoa, Italy.
Francesca Bovis	Department of Health Sciences, Section of Biostatistics, University of Genova, Italy
Francesca Caleri	MS Center, Department of Neurology, F. Tappeiner Hospital Meran (BZ), Italy
Gabriele Siciliano	Department of Clinical and Experimental Medicine, Neurology Unit, University of Pisa, Italy
Gaia Cola	Multiple Sclerosis Clinical and Research Unit, Department of Systems Medicine, Tor Vergata University and Hospital, Rome, Italy
Germana Perego	SC Neurologia ASL 4 Chiavarese
Giacomo Lus	Centro Sclerosi Multipla, II Clinica Neurologica, Università della Campania Luigi Vanvitelli
Giampaolo Brichetto	AISM Rehabilitation Center, Genoa, Italy
Giancarlo Icardi	IRCCS San Martino Hospital, Genoa, Italy. Department of Health Sciences, University of Genoa, Genoa, Italy.
gianmarco bellucci	center for experimental neurological therapies (centers), department of neurosciences, mental health and sensory organs, sapienza university of rome, italy
Giorgio Da Rin	Laboratory Medicine, IRCCS Ospedale Policlinico San Martino, Genova, Italy.
Girolama Alessandra Marfia	Multiple Sclerosis Clinical and Research Unit, Department of Systems Medicine, Tor Vergata University and Hospital, Rome, Italy Neurology Unit, IRCCS NEUROMED, Pozzilli, IS, Italy
Giulia Vazzoler	UOC Neurologia e Centro SM Fondazione Istituto G. Giglio, CefalÃ¹
Giuseppe Liberatore	Neuromuscular and Neuroimmunology Service, IRCCS Humanitas Research Hospital, Rozzano, Italy
Giuseppe Trivelli	SC Neurologia ASL 4 Chiavarese
Graziella Callari	UOC Neurologia e Centro SM Fondazione Istituto G. Giglio, Cefalù
Ilaria Gandoglia	Neurology Unit, Galliera Hospital
Ilaria Maietta	Department of Health Sciences, Section of Biostatistics, University of Genova, Italy
Irene Schiavetti	Department of Health Sciences, Section of Biostatistics, University of Genova, Italy
Jessica Frau	Centro Sclerosi Multipla Ospedale Binaghi Cagliari - ATS Sardegna, Università di Cagliari
Laura Sticchi	Department of Health Sciences (Dissal), University of Genoa, Genoa, Italy. Hygiene Unit, IRCCS Policlinico San Martino Hospital, Genoa, Italy.
Livia Pasquali	Department of Clinical and Experimental Medicine, Neurology Unit, University of Pisa, Italy
Lorena Lorefice	Centro Sclerosi Multipla Ospedale Binaghi Cagliari - ATS Sardegna, Università di Cagliari
Luca Carmisciano	Department of Health Sciences, Section of Biostatistics, University of Genova, Italy
Lucia Ruggiero	Clinica Neurologica, DSNRO Università Federico II di Napoli
Marcello Manzino	Divisione di Neurologia, Ospedale San Paolo, Savona
Marco Salvetti	center for Experimental Neurological Therapies (CENTERS), Department of Neurosciences, Mental Health and Sensory Organs, Sapienza University of Rome, Italy IRCCS Istituto Neurologico MediterraneoNeuromed, Pozzilli, Italy
Margherita Monti Bragadin	AISM Rehabilitation Center, Genoa, Italy
maria chiara buscarinu	center for experimental neurological therapies (centers), department of neurosciences, mental health and sensory organs, sapienza university of rome, italy
Maria Gagliardi	Department of Neurosciences, Rehabilitation, Ophthalmology, Genetics, Maternal and Child health, Genova, Italy
Maria Laura Stromillo	Clinica Neurologica e Malattie Neurometaboliche, Universita' degli Studi di Siena
Maria Pia Sormani	Department of Health Sciences, Section of Biostatistics, University of Genova, Italy IRCCS Ospedale Policlinico San Martino, Genova, Italy/Department of Health Sciences, Section of Biostatistics, University of Genova, Italy
Maria Teresa Ferrò	Neuroimmunology, Center for Multiple Sclerosis, Cerobrovascular Department, Neurological Unit, ASST Crema
Maria Teresa Rilla	Department of Neurology, Imperia Hospital, Imperia, Italy
Marinella Clerico	Dipartimento di Scienze Cliniche e Biologiche, Università di Torino Università di Torino
Mario Alberto Battaglia	Research Department, Italian Multiple Sclerosis Foundation, Genoa, Italy Department of Life Sciences, University of Siena, Siena, Italy
Marta Ponzano	Department of Health Sciences, Section of Biostatistics, University of Genova, Italy
Marzia Fronza	Centro Sclerosi Multipla Ospedale Binaghi Cagliari - ATS Sardegna, Università di Cagliari
Massimo Del Sette	Neurology Unit, Galliera Hospital
Matilde Inglese	Department of Neuroscience, Rehabilitation, Ophthalmology, Genetics, Maternal and Child Health (DINOGMI) and Center of Excellence for Biomedical Research (CEBR), University of Genoa, Genoa, Italy IRCCS Ospedale Policlinico San Martino, Genova, Italy
Matteo Scialabba	U.O. Neurologia e Centro Sclerosi Multipla - Fondazione Istituto G. Giglio Cefalù (PA)
Michele Bedognetti	Centro Sclerosi Multipla S.C. Neurologia Asl 3 Genovese
Monica Ulivelli	Department of Medicine, Surgery and Neuroscience, University of Siena
Nicola De Rossi	Centro Sclerosi Multipla ASST Spedali Civili di Brescia
Nicola De Stefano	Clinica Neurologica e Malattie Neurometaboliche, Università degli Studi di Siena
Paola Gazzola	Centro Sclerosi Multipla S.C. Neurologia Asl 3 Genovese
rachele bigi	center for experimental neurological therapies (centers), department of neurosciences, mental health and sensory organs, sapienza university of rome, italy
Raffaele Dubbioso	Clinica Neurologica, DSNRO Università Federico II di Napoli
roberta reniè	center for experimental neurological therapies (centers), department of neurosciences, mental health and sensory organs, sapienza university of rome, italy
Rosa Iodice	Clinica Neurologica, DSNRO Università Federico II di Napoli
Sabrina Fabbri	Centro Sclerosi Multipla S.C. Neurologia Asl 3 Genovese
Sarah Rasia	Centro Sclerosi Multipla ASST Spedali Civili di Brescia
Simona Rolla	Dipartimento di Scienze Cliniche e Biologiche, Università di Torino Università di Torino
Stefan Platzgummer	Laboratory of Clinical Pathology, F. Tappeiner Hospital Meran (BZ), Italy
Susanna Cordera	Department of Neurology, Ospedale Regionale, Aosta, Italy
Tiziana Tassinari	S.C. Neurologia - Ospedale Santa Corona Pietra Ligure (Sv)
Valentina Carlini	Centro Sclerosi Multipla S.C. Neurologia Asl 3 Genovese

## Contributors

All authors participated in the study planning. Maria Pia Sormani, Matilde Inglese, Irene Schiavetti, Luca Carmisciano, Alice Laroni, Caterina Lapucci, Diego Franciotta, Carlo Serrati, Marco Salvetti, Antonio Uccelli had a role in the study conceptualization and in writing the original draft. Cinzia Cordioli, Doriana Landi, Marinella Clerico, Elisabetta Signoriello, Jessica Frau, Ilaria Gandoglia, Tiziana Tassinari, Germana Perego, Giampaolo Brichetto, Paola Gazzola, Antonio Mannironi, Maria Laura Stromillo, Teresa Ferrò, Alessia Di Sapio, Livia Pasquali, Monica Ulivelli, Fabiana Marinelli, Graziella Callari, Rosa Iodice, Giuseppe Liberatore, Francesca Caleri, Anna Maria Repice, Susanna Cordera had a role in data curation and in writing, reviwing and editing the manuscript. Giorgio da Rin and Diego Franciotta performed formal analyses of blood samples. Mario Alberto Battaglia had a role in project administration and in funding acquisition. Maria Pia Sormani, Irene Schiavetti, Luca Carmisciano had a role in the data curation and statistical analysis. All authors read and approved the final version of the manuscript.

## Data sharing statement

Data can be shared by the authors upon request.

## Declaration of Competing Interest

Caleri F received personal compensations from Merck, Biogen, Genzyme, Roche, Teva. Clerico M received grants and consulting fees from Merck, Biogen, Novartis, Sanofi-Genzyme, Roche, Almirall. Cordioli C received personal compensations from Merck, Biogen, Novartis, Roche, Almirall. Di Sapio A received consulting fees from Novartis, Biogen, Genzyme. Franciotta D received personal honoraria from Merck, Biogen, Sanofi-Genzyme, Roche. Frau J received consulting fees from Biogen, Sanofi-Genzyme, Almirall. Inglese received consulting fees from Merck, Biogen, Novartis, Sanofi-Genzyme, Roche. Iodice received personal honoraria from Merck, Biogen, Sanofi-Genzyme, Roche. Landi received consulting fees from Merck, Biogen, Novartis, Sanofi-Genzyme, Roche, Teva, Mylan, Bristol-Celgene. Laroni received consulting fees from Merck, Biogen, Novartis, Sanofi-Genzyme, Roche. Salvetti received consulting fees from Merck, Biogen, Novartis, Sanofi-Genzyme, Roche. Serrati received grants from Neopharmed. Sormani received consulting fees from Merck, Biogen, Novartis, Sanofi-Genzyme, Roche, Celgene, Geneuro, GSK, Medday, Immunic. Uccelli participated in advisory boards for Biogen, Roche. Ulivelli received consulting fees from Biogen, Novartis, Serono. Battaglia MA, Brichetto G, Callari G, Carmisciano L, Cordera S, Da Rin G, Ferrò MT, Gandoglia I, Gazzola P Lapucci C, Liberatore G, Mannironi A, Marinelli F, Pasquali L, Perego G, Repice AM, Schiavetti I, Signoriello E, Stromillo ML, Tassinari T, have nothing to disclose.

## Appendix Supplementary materials

Image, application 1

Image, application 2
