# Supplementary material for: Effect of SARS-CoV-2 mRNA vaccination in MS patients treated with disease modifying therapies
Source: eBioMedicine. 2021 Sep 22;72:103581. doi: 10.1016/j.ebiom.2021.103581 (PMC8456129; doi:10.1016/j.ebiom.2021.103581)
Supplement: Supplementary file 1 [file mmc1.pdf]

OSPEDALE POLICLINICO SAN MARTINO
Sistema Sanitario Regione Liguria
Istituto di Ricovero e Cura a Carattere Scientifico

June 30th, 2021

To whom it may concern:

We express our agreement to be acknowledged in the Acknowledgments section of the paper: **"Effect of SARS-CoV-2 mRNA vaccination in MS patients treated with disease modifying therapies".**

Barbara Uggeri
Laboratory Medicine,
IRCCS Osp.Policlinico San Martino,
Genova, Italy

Claudio Spallarossa
Laboratory Medicine,
IRCCS Osp.Policlinico San Martino,
Genova, Italy

Giovanni Rossi
Laboratory Medicine,
IRCCS Osp.Policlinico San Martino,
Genova, Italy
