# Supplementary material for: Effect of SARS-CoV-2 mRNA vaccination in MS patients treated with disease modifying therapies
Source: eBioMedicine. 2021 Sep 22;72:103581. doi: 10.1016/j.ebiom.2021.103581 (PMC8456129; doi:10.1016/j.ebiom.2021.103581)
Supplement: Supplementary file 2 [file mmc2.docx]

| Authors | Affiliation |
| --- | --- |
| Alessandro Maglione | Dipartimento di Scienze Cliniche e Biologiche, Università di Torino Università di Torino |
| Alessia Di Sapio | Department of Neurology, Regina Montis Regalis Hospital, Mondovì, Italy |
| Alessio Signori | Department of Health Sciences, Section of Biostatistics, University of Genova, Italy |
| Alice Laroni | Department of Neuroscience, Rehabilitation, Ophthalmology, Genetics, Maternal and Child Health (DINOGMI) and Center of Excellence for Biomedical Research (CEBR), University of Genoa, Genoa, Italy IRCCS Ospedale Policlinico San Martino, Genova, Italy |
| Aniello Iovino | Clinica Neurologica, DSNRO Università  Federico II di Napoli |
| Anna Maria Repice | Department of Neurology 2, Careggi University Hospital, Florence, Italy |
| Antonio Mannironi | Department of Neurology, Sant'Andrea Hospital, La Spezia, Italy |
| Antonio Uccelli | Department of Neuroscience, Rehabilitation, Ophthalmology, Genetics, Maternal and Child Health (DINOGMI) and Center of Excellence for Biomedical Research (CEBR), University of Genoa, Genoa, Italy IRCCS Ospedale Policlinico San Martino, Genova, Italy |
| Carlo Serrati | Department of Neurology, Imperia Hospital, Imperia, Italy |
| Carolina Gabri Nicoletti | Multiple Sclerosis Clinical and Research Unit, Department of Systems Medicine, Tor Vergata University and Hospital, Rome, Italy |
| Caterina Lapucci | Department of Neuroscience, Rehabilitation, Ophthalmology, Genetics, Maternal and Child Health (DINOGMI) and Center of Excellence for Biomedical Research (CEBR), University of Genoa, Genoa, Italy IRCCS Ospedale Policlinico San Martino, Genova, Italy |
| Chiara Rosa Mancinelli | Centro Sclerosi Multipla ASST Spedali Civili di Brescia |
| Cinzia Cordioli | Centro Sclerosi Multipla ASST Spedali Civili di Brescia |
| Daiana Bezzini | Department of Life Sciences, University of Siena |
| Daniele Carmagnini | Centro Sclerosi Multipla Ospedale Binaghi Cagliari - ATS Sardegna, Università  di Cagliari |
| Davide Brogi | S.C. Neurologia - Ospedale Santa Corona |
| Diego Franciotta | Autoimmunology Laboratory, IRCCS Ospedale Policlinico San Martino, Genoa, Italy |
| Doriana Landi | Multiple Sclerosis Clinical and Research Unit, Department of Systems Medicine, Tor Vergata University and Hospital, Rome, Italy |
| Eduardo Nobile Orazio | Neuromuscular and Neuroimmunology Service, IRCCS Humanitas Research Hospital, Rozzano, Italy Department of Medical Biotechnology and Translational Medicine, Milan University, Milan, Italy |
| Eleonora Cocco | Centro Sclerosi Multipla Ospedale Binaghi Cagliari - ATS Sardegna, Università  di Cagliari |
| Elisabetta Signoriello | Centro Sclerosi Multipla, II Clinica Neurologica, Università  della Campania Luigi Vanvitelli |
| Enri Nako | Department of Neurology, Regina Montis Regalis Hospital, MondovÃ¬, Italy |
| Ester Assandri | Neuroimmunology, Center for Multiple Sclerosis, Cerobrovascular Department, Neurological Unit, ASST Crema |
| Fabiana Marinelli | Multiple Sclerosis Center, Fabrizio Spaziani Hospital, via Armando Fabi, Frosinone – Italy |
| Federica Baldi | Department of Neurosciences, Rehabilitation, Ophthalmology, Genetics, Maternal and Child health, University of Genova, Genova, Italy |
| Filippo Ansaldi | Planning, Epidemiology and Prevention Unit, A.Li.Sa. Liguria Health Authority, Genoa, Italy.  IRCCS San Martino Hospital, Genoa, Italy.  Department of Health Sciences, University of Genoa, Genoa, Italy. |
| Francesca Bovis | Department of Health Sciences, Section of Biostatistics, University of Genova, Italy |
| Francesca Caleri | MS Center, Department of Neurology, F. Tappeiner Hospital Meran (BZ), Italy |
| Gabriele Siciliano | Department of Clinical and Experimental Medicine, Neurology Unit, University of Pisa, Italy |
| Gaia Cola | Multiple Sclerosis Clinical and Research Unit, Department of Systems Medicine, Tor Vergata University and Hospital, Rome, Italy |
| Germana Perego | SC Neurologia ASL 4 Chiavarese |
| Giacomo Lus | Centro Sclerosi Multipla, II Clinica Neurologica, Università  della Campania Luigi Vanvitelli |
| Giampaolo Brichetto | AISM Rehabilitation Center, Genoa, Italy |
| Giancarlo Icardi | IRCCS San Martino Hospital, Genoa, Italy.  Department of Health Sciences, University of Genoa, Genoa, Italy. |
| Gianmarco Bellucci | Centre for Experimental Neurological Therapies (CENTERS), Department of Neurosciences, Mental Health and Sensory Organs, Sapienza University of Rome, Italy |
| Giorgio Da Rin | Laboratory Medicine, IRCCS Ospedale Policlinico San Martino, Genova, Italy. |
| Girolama Alessandra Marfia | Multiple Sclerosis Clinical and Research Unit, Department of Systems Medicine, Tor Vergata University and Hospital, Rome, Italy  Neurology Unit, IRCCS NEUROMED, Pozzilli, IS, Italy |
| Giulia Vazzoler | UOC Neurologia e Centro SM Fondazione Istituto G. Giglio, CefalÃ¹ |
| Giuseppe Liberatore | Neuromuscular and Neuroimmunology Service, IRCCS Humanitas Research Hospital, Rozzano, Italy |
| Giuseppe Trivelli | SC Neurologia ASL 4 Chiavarese |
| Graziella Callari | UOC Neurologia e Centro SM Fondazione Istituto G. Giglio, Cefalù |
| Ilaria Gandoglia | Neurology Unit, Galliera Hospital |
| Ilaria Maietta | Department of Health Sciences, Section of Biostatistics, University of Genova, Italy |
| Irene Schiavetti | Department of Health Sciences, Section of Biostatistics, University of Genova, Italy |
| Jessica Frau | Centro Sclerosi Multipla Ospedale Binaghi Cagliari - ATS Sardegna, Università di Cagliari |
| Laura Sticchi | Department of Health Sciences (Dissal), University of Genoa, Genoa, Italy.  Hygiene Unit, IRCCS Policlinico San Martino Hospital, Genoa, Italy. |
| Livia Pasquali | Department of Clinical and Experimental Medicine, Neurology Unit, University of Pisa, Italy |
| Lorena Lorefice | Centro Sclerosi Multipla Ospedale Binaghi Cagliari - ATS Sardegna, Università  di Cagliari |
| Luca Carmisciano | Department of Health Sciences, Section of Biostatistics, University of Genova, Italy |
| Lucia Ruggiero | Clinica Neurologica, DSNRO Università  Federico II di Napoli |
| Marcello Manzino | Divisione di Neurologia, Ospedale San Paolo, Savona |
| Marco Salvetti | Centre for Experimental Neurological Therapies (CENTERS), Department of Neurosciences, Mental Health and Sensory Organs, Sapienza University of Rome, Italy  IRCCS Istituto Neurologico Mediterraneo Neuromed, Pozzilli, Italy |
| Margherita Monti Bragadin | AISM Rehabilitation Center, Genoa, Italy |
| Maria Chiara Buscarinu | Centre for Experimental Neurological Therapies (CENTERS), Department of Neurosciences, Mental Health and Sensory Organs, Sapienza University of Rome, Italy |
| Maria Gagliardi | Department of Neurosciences, Rehabilitation, Ophthalmology, Genetics, Maternal and Child health, Genova, Italy |
| Maria Laura Stromillo | Clinica Neurologica e Malattie Neurometaboliche, Universita' degli Studi di Siena |
| Maria Pia Sormani | Department of Health Sciences, Section of Biostatistics, University of Genova, Italy IRCCS Ospedale Policlinico San Martino, Genova, Italy/Department of Health Sciences, Section of Biostatistics, University of Genova, Italy |
| Maria Teresa Ferrò | Neuroimmunology, Center for Multiple Sclerosis, Cerobrovascular Department, Neurological Unit, ASST Crema |
| Maria Teresa Rilla | Department of Neurology, Imperia Hospital, Imperia, Italy |
| Marinella Clerico | Dipartimento di Scienze Cliniche e Biologiche, Università di Torino Università di Torino |
| Mario Alberto Battaglia | Research Department, Italian Multiple Sclerosis Foundation, Genoa, Italy  Department of Life Sciences, University of Siena, Siena, Italy |
| Marta Ponzano | Department of Health Sciences, Section of Biostatistics, University of Genova, Italy |
| Marzia Fronza | Centro Sclerosi Multipla Ospedale Binaghi Cagliari - ATS Sardegna, Università  di Cagliari |
| Massimo Del Sette | Neurology Unit, Galliera Hospital |
| Matilde Inglese | Department of Neuroscience, Rehabilitation, Ophthalmology, Genetics, Maternal and Child Health (DINOGMI) and Center of Excellence for Biomedical Research (CEBR), University of Genoa, Genoa, Italy IRCCS Ospedale Policlinico San Martino, Genova, Italy |
| Matteo Scialabba | U.O. Neurologia e Centro Sclerosi Multipla - Fondazione Istituto G. Giglio Cefalù (PA) |
| Michele Bedognetti | Centro Sclerosi Multipla S.C. Neurologia Asl 3 Genovese |
| Monica Ulivelli | Department of Medicine, Surgery and Neuroscience, University of Siena |
| Nicola De Rossi | Centro Sclerosi Multipla ASST Spedali Civili di Brescia |
| Nicola De Stefano | Clinica Neurologica e Malattie Neurometaboliche, Università degli Studi di Siena |
| Paola Gazzola | Centro Sclerosi Multipla S.C. Neurologia Asl 3 Genovese |
| Rachele Bigi | Centre for Experimental Neurological Therapies (CENTERS), Department of Neurosciences, Mental Health and Sensory Organs, Sapienza University of Rome, Italy |
| Raffaele Dubbioso | Clinica Neurologica, DSNRO Università  Federico II di Napoli |
| Roberta Reniè | Centre for Experimental Neurological Therapies (CENTERS), Department of Neurosciences, Mental Health and Sensory Organs, Sapienza University of Rome, Italy |
| Rosa Iodice | Clinica Neurologica, DSNRO Università  Federico II di Napoli |
| Sabrina Fabbri | Centro Sclerosi Multipla S.C. Neurologia Asl 3 Genovese |
| Sarah Rasia | Centro Sclerosi Multipla ASST Spedali Civili di Brescia |
| Simona Rolla | Dipartimento di Scienze Cliniche e Biologiche, Università di Torino Università di Torino |
| Stefan Platzgummer | Laboratory of Clinical Pathology, F. Tappeiner Hospital Meran (BZ), Italy |
| Susanna Cordera | Department of Neurology, Ospedale Regionale, Aosta, Italy |
| Tiziana Tassinari | S.C. Neurologia - Ospedale Santa Corona Pietra Ligure (Sv) |
| Valentina Carlini | Centro Sclerosi Multipla S.C. Neurologia Asl 3 Genovese |
